# Predictors of Long-Term Outcomes of Video-Laparoscopic Versus Open Surgery in Obese Patients with Colorectal Cancer: A Propensity Score Matching Study

**DOI:** 10.3390/cancers14112669

**Published:** 2022-05-27

**Authors:** Cinzia Bizzoca, Roberta Zupo, Fabio Castellana, Annamaria Sila, Felicia Fiore, Fabrizio Aquilino, Rodolfo Sardone, Leonardo Vincenti

**Affiliations:** 1 General Surgery Unit “Ospedaliera”, University Hospital “Policlinico” of Bari, 70124 Bari, Italy; cinzia.bizzoca@policlinico.ba.it (C.B.); f.fiore27@studenti.uniba.it (F.F.); leonardo.vincenti@policlinico.ba.it (L.V.); 2 Unit of Research Methodology and Data Sciences for Population Health, National Institute of Gastroenterology “Saverio de Bellis”, Research Hospital, Castellana Grotte, 70013 Bari, Italy; roberta.zupo@irccsdebellis.it (R.Z.); fabio.castellana@irccsdebellis.it (F.C.); annamaria.sila@irccsdebellis.it (A.S.); 3 General Surgery Unit, National Institute of Gastroenterology “Saverio de Bellis”, Research Hospital, Castellana Grotte, 70013 Bari, Italy; fabrizio.aquilino@irccsdebellis.it

**Keywords:** colorectal cancer, laparoscopic surgery, open surgery, obesity, long-term outcomes

## Abstract

**Simple Summary:**

Reducing invasiveness in colorectal surgery settings offers advantages, especially in patients with obesity. This study aimed to fill the exploratory window on the long-term oncological safety of the video-laparoscopic versus open colorectal surgical approach in obesity. A retrospective analysis of a surgical database of 138 colorectal cancer patients was performed by applying propensity score matching as a way to reduce selection bias. Overall survival and cancer-free survival were chosen as the primary long-term outcomes to compare open and video-laparoscopic surgical approaches, in order to enrich the body of evidence on the topic and provide greater confidence in clinical settings.

**Abstract:**

**Background:** Minimally invasive methods in colorectal surgery offer unquestionable advantages, especially in the context of obesity. The current study addresses the lack of scientific evidence on the long-term oncologic safety of video-laparoscopic (VL) approaches in excess-weight CRC patients undergoing surgery. **Methods:** We retrospectively analyzed a surgical database consisting of 138 CRC patients undergoing VL (*n* = 87, 63%) and open CRC surgery (*n* = 51, 37%). To reduce selection bias, a propensity score matching was applied as a preliminary step to balance the comparison between the two surgery groups, i.e., VL and open surgery. Data from patients treated by the same surgeon were used.to minimize bias. Additional Cox regression models were run on the matched sample (N = 98) to explore the observed benefits of VL surgery in terms of overall and cancer-free survival. The nonparametric Kaplan-Meier method was used to compare the two surgical approaches and assess the likelihood of survival and cancer relapse. **Results**: The study sample was mostly male (N = 86, 62.3%), and VL outnumbered open surgery (63% versus 37%). Both before and after the matching, the VL-allocated group showed better overall survival (*p* < 0.01) with comparable cancer-free survival over more than five years of median observation time (66 months). Kaplan Meier survival probability curves corroborated the VL significant protective effect on survival (HR of 0.32; 95% CI: 0.13 to 0.81) even after adjusting for major confounding factors (age, gender, comorbidity index, BMI, tumor localization, tumor staging, tumor grading, clearance, CRM). Findings on oncologic performance by tumor relapse were comparable but lacked significance due to the small number of events observed. **Conclusions:** Comparing CRC surgical approaches, VL allocation showed comparable cancer-free survival but also a better performance on overall mortality than open surgery over more than five years of median observation.

## 1. Introduction

Latest metrics skew 13% (11% of men and 15% of women) on the global prevalence of excess weight people, peaking at 39% when looking at the adult population subgroup (precisely, 39% of males and 40% of females). In other words, between 1975 and 2016, obesity phenotypes increased about thrice globally. Research efforts around the biological pathways and chronic health complications associated with obesity show no sign of stopping but rather are currently a susceptible topic [1]. Moving back towards leading risk drivers of chronic non-communicable diseases, analysis of excess weight phenotypes has long been suggested as a useful prognostic indicator for colorectal cancer (CRC), as body weight appears to be a prognostic marker to follow in a preventive setting. CRC is the third most common malignancy and the fourth leading cause of death globally to date, and a recent meta-analysis report demonstrated a 30% increased risk of CRC in males and a 12% increased risk in females for each 5-unit increase in BMI [2]. Looking at pathophysiology, baseline inflammation is increased in individuals with obesity, and the current doctrine in obesity-related cancer research is that excess visceral adiposity generates adipocytokines that power a proinflammatory state with an associated pro-oncogenic and prometastatic environment [3].

CRC surgery is very challenging, and success may be compromised even when performed by highly skilled surgeons. Even more, fat overweight phenotypes represent a subpopulation at increased risk for high technical difficulties during video laparoscopic (VL) colorectal procedures, often resulting in conversion to open surgery [4,5]. On the other hand, VL compared with open colorectal surgery is recognized as associated with reduced postoperative pain, shorter hospital stay, less disability, and reduced pulmonary dysfunction [6]. This way, our recent report on obesity phenotypes undergoing CRC surgery further demonstrated the safe and favorable short-term outcome of the minimally invasive approach compared to the open approach when looking at the postoperative length of stay and severity of postoperative complications [7].

Nevertheless, there is still a need for consistency in the data in the literature regarding the information on long-term VL surgery results in obesity CRC phenotypes. This topic is of particular interest as many questions have been raised about the oncologic safety of the minimally invasive approach to colorectal surgery in this kind of patient. Special attention has been paid to the number of lymph nodes recovered and the achievement of adequate histologic margins when VL surgery is compared with the standard open approach [4,8].

Our study aimed to evaluate the impact of the VL approach versus open surgery on long-term survival and cancer relapse risks on average five years of observation in a group of obesity phenotypes affected by CRC after surgery.

## 2. Materials and Methods

### 2.1. Study Population and Design

Between January 2013 and December 2020, 138 consecutive CRC patients were scheduled for laparoscopic or open resection by the same senior surgeon (L.V.) in two different surgical units, namely the University Hospital “Policlinico” of Bari (Apulia, Southern Italy) and the National Institute of Gastro-enterological Research Hospital “Saverio de Bellis” (Castellana Grotte, Apulia, Southern Italy). The patients were chosen using a non-probabilistic convenience sampling method.

The treatment plan followed the guidelines of the National Comprehensive Cancer Network (NCCN) [9]. The patients had to satisfy the following criteria: a confirmed diagnosis of colorectal cancer, a pre-operative state of obesity (i.e., a BMI of 30 kg/m^2^), and a minimum age of 18 years at the time of recruitment. Patients with concurrent emergency conditions (such as perforation and/or occlusion), pregnancy, co-existing peritoneal carcinomatosis, combined operations for other diseases, contraindications to VL surgery (such as NYHA class IV cardiopathy, severe chronic obstructive pulmonary disease), and the need for transverse resection or total colectomy were all ruled out. After the lead surgeon’s (LV) assessment of the surgical challenge, the decision to perform open or VL surgery for each patient was based on the pre-operative anesthesiologic risk (i.e., physical evaluation according to the American Society of Anesthesiologists (ASA) criteria), which was clinically assessed by an experienced Intensive Care Unit (ICU) specialist. Because of the substantial experience in colorectal surgery required for this kind of intervention, all procedures were performed by the same lead surgeon (LV) to decrease the risk of operator bias. The Ethics Committee of the National Institute of Gastroenterology “S. De Bellis” Research Hospital approved the research protocol (Protocol n. 234/2019, ClinicalTrials.gov Identifier: NCT04716062), which followed the principles of the Declaration of Helsinki (Castellana Grotte, Apulia, Italy). To participate in this research, all patients provided their written or verbal agreement.

### 2.2. Demographic and Clinical Variables

Electronic and paper medical records were used to obtain clinical data. Age, gender, body weight and height, comorbidities, and BMI were all included in the database (BMI). A senior nutritionist (RZ) measured participants in light clothing and no shoes. All variables were collected between 7:00 and 10:00 a.m. after an overnight fast. A wall-mounted stadiometer measured height to 0.5 cm (Seca 711; Seca, Hamburg, Germany). A calibrated balance beam scale was used to calculate body weight to the closest to 0.1 kg (Seca 711; Seca, Hamburg, Germany). Body mass index (BMI) was computed by multiplying body weight (kg) by the square of height (m^2^), and obesity was defined by the World Health Organization (≥30.0 kg/m^2^) [10]. We recorded tumor location (right, left, or rectum), tumor stage (I–IV) and grading (G1–G3), anastomosis (yes/no), previous abdominal surgery (yes/no), specimen length (cm), operative time (min), harvested lymph nodes (n), and distal clearance (mm) [11,12]. Based on the AJCC/TNM classification [12], tumor stage was defined according to the combination of three parameters, described as follows: T1 tumor invading submucosa, T2 tumor invading muscularis propria, T3 tumor invading through the muscularis propria into peri-colorectal tissues, T4 tumor penetrating the surface of the visceral peritoneum or tumor directly invading or adherent to surrounding organs or structures; N0 no lymph node invasion, N1 tumoral invasion of 1 to 3 lymph nodes; N2 tumoral invasion of 4 or more lymph nodes; M0 no distant metastasis, M1 distant or peritoneal metastasis. Four stages (UICC categories) with different prognosis were identified: stage I T1-2, N0M0; stage II T3-4 N0-M0, stage III T1/4-N1/2-M0, stage IV any T any N M1. Histopathological criteria required an experienced pathologist to rate the malignancy using an incremental score (G1–G3) [13].

### 2.3. Long-Term Postoperative Outcomes

Primary outcome was overall survival. To this end, patient death was recorded and overall survival was calculated as months passed from the intervention to the exitus. The secondary outcome was cancer-free survival, intended as months from the intervention to the cancer relapse (when occurred). All data were recorded retrospectively.

### 2.4. Surgical Procedures

*Right colectomy.* The Verres needle was inserted in the left subcostal location and three trocars were put to achieve pneumoperitoneum at 12–14 mmHg. A complete mesocolic excision was always carried out. To ensure proper lymphadenectomy, all surgeries started with closing the ileocolic artery near the origin of the mesenteric axis. Hepatic flexure tumors were treated with partial omentectomy and central closure of the gastroepiploic artery. To restore bowel continuity, an intracorporeal ileo-transverse mechanical anastomosis was performed. A Pfannenstiel mini-incision was used to extract the specimen, which was protected with an endobag or steri-drape.

Through a midline laparotomy, open procedures were performed using the same technical rules.

*Left colectomy.* The laparoscopic procedure also required a pneumoperitoneum of 12–14 mmHg, which was obtained applying the Hasson technique. Depending on the abdomen form, three or four trocars were utilized to ensure adequate exposure of surgical landmarks. To allow for a “floppy” anastomosis, the left colic flexure was often mobilized first. A complete mesocolic excision was always carried out. The inferior mesenteric vein was identified and ligated to the pancreas’s inferior border. The inferior mesenteric artery was ligated at the aortic plane, with careful preservation of the hypogastric nerves. An end-to-end transanal colorectal anastomosis was created using the Knight-Griffen approach [14]. Open procedures were performed with the same technical rules through a midline laparotomy.

*Rectal resection.* From the insertion of four trocars to left colon mobilization and vascular ligations, the early phases of a laparoscopic rectal resection were comparable to those of a left colectomy. Upper rectal cancers were treated with partial mesorectal excision (PME), whereas middle and lower rectal tumors were treated with complete mesorectal excision (TME) [15]. When coloanal anastomosis was necessary, the anastomosis was done manually or using the Knight-Griffen procedure [16]. In the event of coloanal anastomosis and complete mesorectal excision (TME) in patients with comorbidities and/or previous neoadjuvant treatment, an ileostomy was usually performed. In patients with many comorbidities, the Hartmann operation has been used to treat locally advanced rectal cancer [17]. Only ultra-low rectal neoplasia with sphincter invasion has been treated with the Miles method [18].

### 2.5. Statistical Analysis

The baseline variables were statistically analyzed and expressed as mean standard deviation (SD) for continuous variables, and proportion (percent) for categorical variables. Shapiro’s test is used to test the normality of the distribution for each variable. To eliminate collinearity effects in the model, Spearman’s correlation matrix was constructed for all continuous pathological and anthropometric variables to control for associated variables.

Numerous null hypothesis significance tests were used to compare the VL and open surgery groups: For non-normally distributed continuous variables, the Mann Whitney sum rank test was used; For normally distributed continuous variables, the independent samples *t*-test was used; for categorical variables, the chi-squared test was used; and for categorical variables with fewer than five observations, the Fisher’s exact test was used. *p*-values less than or equal to 0.05 with 95% confidence intervals were considered statistically significant.

A propensity score model was developed to balance group comparisons and minimize selection bias caused by patient arbitrary surgical allocation. The propensity index was obtained by regressing treatment status (VL) on baseline parameters. The estimated propensity score was derived from the fitted regression model’s predicted likelihood of treatment.

Nearest Neighbour (NN) Analysis was performed to compare patients in the intervention group (VL) to those in the control group (open surgery) [19], after controlling for the primary factors of age, gender, BMI, and tumor location (right, left, or rectum) and using a caliper of 0.1. After the groups were matched, we ran a diagnostic balancing analysis to see how well they matched. We opted to run regression models in which we additionally adjusted the impact for the same covariates of matching variables because the balance of matching was not ideal (one to one) for each variable [20].

To investigate relationships with long-term outcome factors, two multivariable Cox regression models were run: the dependent variables were overall disease-free mortality and time to tumor relapse. We created three hierarchical nested models, each adjusted for a different set of main confounders, to assess the relationship between treatment variables and outcomes regardless of other factors that may affect the effect: (1) unprocessed model using simply VL as a covariate (2) model 1 plus age, gender, and BMI; (3) model 2 plus tumor site; length of the specimen, prior surgery, staging, clearance/CRM, harvested lymph nodes, and stoma position confounding covariates were chosen based on the traditional definition of confounders, which states that they must be linked to both exposure (i.e., surgical treatment) and outcome. The Kaplan-Meier approach was used to determine the likelihood of survival between enrollment in a study and the occurrence of a subsequent event. The log-rank test was performed to examine if the two groups’ survival rates were comparable. A senior epidemiologist (R.S.) and a biostatistician (F.C.) designed and implemented the methodological approach and analyses using the RStudio program, version 1.2.5042.

## 3. Results

**Figure 1:**
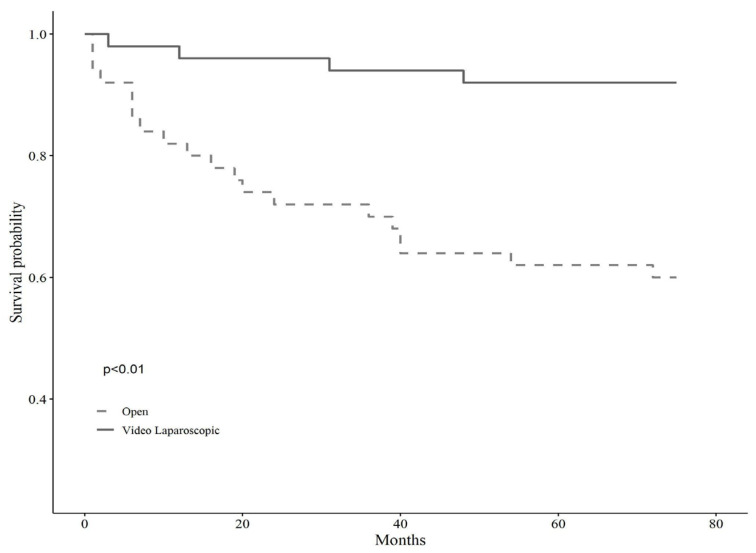
Kaplan Meir mortality curves according to the type of surgery.

**Figure 2:**
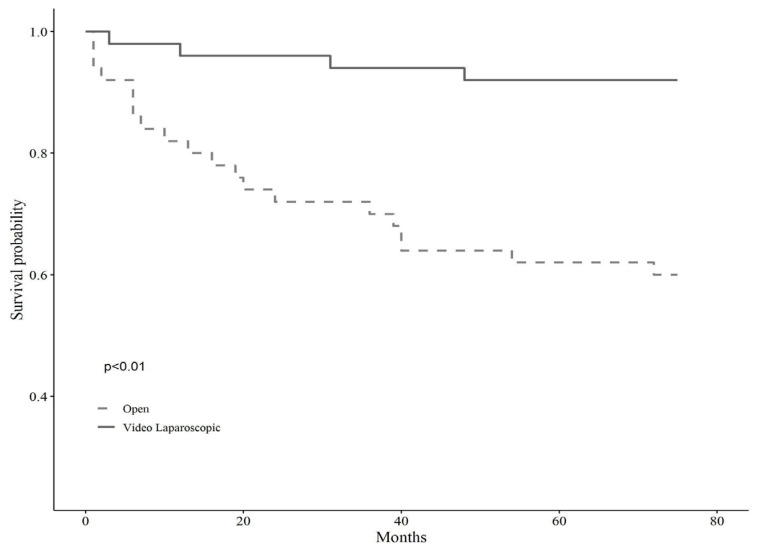
Kaplan Meir curves on relapse of tumor according to the type of surgery.

A detailed description of the entire sample according to surgical treatment before and after matching is shown in Table 1. The baseline sample was 138 patients, 51 of whom were assigned to open surgery (37%, N = 51) and 87 to VL (63%, N = 87). After applying the propensity score modeling, we matched 1:1 on the nearest neighbor estimate to obtain two balanced groups (50% open surgery vs. 50% VL, N = 102). Before the matching, the gender balance was 49% female and 51% male for the open surgery group, whereas 19.60% female and 80.40% male for the VL surgery group. The mean age was shown to be statistically different across groups, being 72 ± 9.02 years for open surgery and 66.83 ± 10.2 for VL, as well as the BMI, being 35.13 ± 5.45 kg/m^2^ for the open surgery group and 31.99 ± 2.36 kg/m^2^ for the VL group (*p* < 0.001). Tumor localization showed no significant differences across groups before matching (*p* = 0.10). However, left localization was trending in subjects assigned to VL surgery (35.6% vs. 19.6%), while the right localization prevailed in those assigned to open surgery (37.30% vs. 25.3%). Tumor differentiation grading showed significant differences across groups (*p* < 0.01), such that grade G3, i.e., poorly differentiated, was more represented in the open surgery group (34.1% vs. 9%), while grade G2, i.e., moderately differentiated, was predominant in VL surgery (70.50% vs. 43.90%). Postoperative hospitalization time differed significantly between the two groups, either before or after matching, with fewer days for subjects allocated to VL surgery, and the Clavien-Dindo post-operative severity classification system showed no differences across surgery groups. Anyhow, the short-term results of the same sample have been already discussed elsewhere [7]. Lastly, the observation time, i.e., follow-up, proved to be significantly longer for subjects undergoing VL surgery, predicting better long-term outcomes (*p* < 0.01). Kaplan Meir mortality curves showed markedly reduced survival in subjects who underwent open surgery (*p* < 0.01) over about five years of observation time (66 months) (Figure 1); conversely, tumor relapse was more frequent in those receiving VL surgery, but this finding lacked statistical significance (*p* 0.45) (Figure 2).

A propensity score matching balance table is included in Table 2.

Table 3 shows the results of multivariate Cox regression analyses using overall death as the dependent variable and three distinct models, all of which were nested hierarchically and adjusted stepwise for the key confounding factors. Because VL intervention was negatively associated with time to follow-up, we regarded it to be the therapy of interest. Even after controlling for age, sex, BMI, and tumor location, our findings indicated that VL-treated patients had a 72% lower chance of dying (HR 0.28, 95% CI 0.10 to 0.99) (right or left). This positive effect improved up to 97% when additional confounding variables, i.e., specimen length, clearance, tumor staging, previous surgery, harvested lymph nodes, and comorbidity score, were fitted into the model (HR 0.03, 95%CI 0.02 to 0.37).

Similarly, multivariate Cox regression analyses were run on time to relapse as the dependent variable with two different models nested hierarchically and adjusted stepwise for key confounding covariates (Table 4). VL surgery was negatively associated with time to tumor relapse, but models, both raw (HR 1.70, 95%CI 0.40 to 7.13), semi- (1.38, 95%CI 0.50 to 38.84), and fully adjusted (HR 1.42, 95%CI 0.42 to 40.00), lacked statistical significance.

## 4. Discussion

The present study was designed to add evidence to the body of literature on the long-term outcome of VL surgery compared with open surgery in obesity CRC phenotypes. To this end, overall survival and cancer-free survival were chosen as the primary long-term outcomes to compare the two surgical approaches.

As primary findings, VL surgery proved to have some “protective effect” on overall survival, when compared with a homogeneous group of excess weight patients operated by open surgery. On the contrary, no significance was reached on tumor relapse after comparing the two surgical approaches.

In a previous case-matched study conducted at our institution [7], we found that VL surgery correlates with better short-term outcomes, in terms of the degree of complication (one point less according to Clavien-Dindo, on average) and length of stay (almost two days shorter). In contrast, we found that fewer harvested lymph nodes were found for the VL group. A variety of parameters, including age, cancer location, neoadjuvant therapy, disease stage, kind of surgery, surgeon and pathologist skill, pathological characteristics, and surgical resection length, are known to influence the number of retrieved lymph nodes [21]. Furthermore, it has been suggested that when the ASA score and BMI rise, the number of lymph nodes decreases [18]. Nonetheless, the average number of lymph nodes taken during CRC surgery in the study cohort was more than 12, above the worldwide standards’ suggestion [19]. Importantly, the laparoscopic group’s mean distal and circumferential margins were comparable to the open surgery group. The overall and cancer-free survival of the patients analyzed were recorded, and the survival curves of the two surgical groups were compared to further investigate the oncological appropriateness of VL surgery in excess weight patients.

The cancer-free survival curves showed a trend in favor of the open surgery group (*p* 0.45). This result has no statistical significance due to the small number of events observed (number of patients with tumor relapse five VL versus three open surgery). Moreover, the relapse cases are few and almost all distant, i.e., metastatic.

On the other hand, the overall survival analysis of the two groups revealed that patients who operated in VL experienced reduced all-cause long-term mortality, on a median follow-up of 66 months. This result was maintained also after matching and multivariate analysis, which were applied to adjust the data for age, sex, BMI, tumor location, stage, grade, and comorbidities.

A systematic review and meta-analysis [4] reported that there was no difference in the oncological outcome of excess- and normal weight-patients operated in VL, confirming the oncological safety of VL surgery in obesity settings.

Instead, this is the first study on a series of excess weight patients affected by CRC, reporting a protective effect of VL surgery on overall survival. This unexpected result could be partially related to better immune status and lower cardiorespiratory and general complications experienced in the postoperative course [7]. On the other hand, the laparoscopic approach seems to have a protective effect on all-cause mortality, especially in frail patients. This effect remains unclear, but it has also been observed in other patient cohorts, such as cirrhotic patients. Indeed, a meta-analysis of more than 4000 patients operated on in VL for hepatocarcinoma showed a reduction in 5-year all-cause mortality [20]. Another systematic review showed that a reduction in postoperative complications has a long-term effect on both OS and DFS [22]. These observations could mean that favorable postoperative outcome, which has been well studied in all fields of laparoscopy, has a positive and not fully explainable influence on long-term outcomes.

It is well-known that VL colorectal surgery is challenging, for two main reasons: for technical surgical difficulties in achieving a satisfying vision as well as an adequate lymphadenectomy; and because of increased risks of complications and the difficult management of comorbid illnesses [4]. For all these reasons, excess weight patients could be defined as “complex” in principle. Probably, the general health status and psychophysical equilibrium were better preserved by the laparoscopic approach in these patients. This could explain the “protective effect” on overall survival that we observed, despite no effect on tumor relapse was determined by the surgical approach.

In conclusion, the VL technique for obesity CRC phenotypes was associated with reduced all-cause mortality in the long term. In this context, Chern et al., found the same performance for VL surgery in a sample of another subset of “complex” patients, that is older subjects. They found there were advantages in terms of shorter hospital stay and oncologic outcomes compared with open surgery, including overall survival and disease-free survival [23]. Then, our meaningless data on tumor relapse, due to the small number of recurrences observed (five VL versus three open surgery), did not allow us to draw any inferences, although the direction of the association seemed unfavorable to the VL approach. In this regard, previous studies have shown similar recurrence rates and sites between the VL and open surgery groups. However, although relapse is a common feature of VL and open surgery for advanced CRC, there is some evidence that the oncologic outcome at a minimum of 2 years is not compromised by the VL approach [21].

There are a few important study limitations to consider. First, there could have been a selection bias because patients were assigned to the VL or open surgery groups based on the ICU and chief surgeon’s subjective clinical judgment. This decision was largely based on each patient’s overall health and the surgeon’s learning curve, so it could have had a significant confounding effect that we were unable to control. Second, patient heterogeneity in terms of underlying clinical characteristics, tumor types and locations, and age may influence outcomes, resulting in a massive residual confounding effect that is impossible to control without careful case selection. Third, the study’s limited sample size represented a weakness. Further, in efforts to minimize bias by including patients operated on by the same surgeon, however, this aspect could be perceived as a limitation to reaching solid conclusions. Then, analyses could not be supplemented with data from postoperative chemotherapy and radiotherapy because of a lack of data. This is the first research, however, to use a double-checked technique for confounders. Indeed, propensity ratings were utilized to eliminate selection bias, and the found VL connection with superior postoperative outcomes was assessed using a full set of variables. Finally, the longitudinal design supports our research setting in terms of the causal interpretation of the findings, despite the limitations of not having data on particular mortality.

## 5. Conclusions

In the present study, VL surgery for CRC in obesity phenotypes performed better than open surgery on overall survival along five years of observation time. However, further studies on larger populations are needed to corroborate these findings.

## Figures and Tables

**Table 1:** Description of the whole sample according to the type of treatment, before and after matching.

	Before Matching					After Matching				
	Open		Video Laparoscopic			Open		Video Laparoscopic		
	Mean ± sd	Median	Mean ± sd	Median	*p*	Mean ± sd	Median	Mean ± sd	Median	*p*
		(Min to Max)		(Min to Max)			(Min to Max)		(Min to Max)	
Prop. (%)	**51 (37.00)**		**87 (63.00)**			**51 (50.00)**		**51 (50.00)**		
Age (years)	72 ± 9.02	73 (45 to 89)	66.83 ± 10.27	68 (34 to 86)	**<0.01**	72 ± 9.02	73 (45 to 89)	61.73 ± 9.15	64 (34 to 76)	**<0.01**
Sex										
*Females*	25 (49.00)		27 (31.00)		**0.03**	25 (49.00)		10 (19.60)		**0.01**
*Males*	26 (51.00)		60 (69.00)			26 (51.00)		41 (80.40)		
BMI (Kg/m^2^)	35.13 ± 5.45	34 (28 to 54.8)	32.98 ± 3.56	32 (28.8 to 45.8)	**0.01**	35.13 ± 5.45	34 (28 to 54.8)	31.99 ± 2.36	31.2 (28.8 to 38.2)	**<0.01**
Location										
*SX*	10 (19.60)		31 (35.60)		0.10	10 (19.60)		22 (43.10)		**0.02**
*DX*	19 (37.30)		22 (25.30)			19 (37.30)		10 (19.60)		
*Rectum*	22 (43.10)		34 (39.10)			22 (43.10)		19 (37.30)		
Grading										
*G1*	9 (22.00)		16 (20.50)		**0.01**	9 (22.00)		10 (20.80)		**<0.01**
*G2*	18 (43.90)		55 (70.50)			18 (43.90)		34 (70.80)		
*G3*	14 (34.10)		7 (9.00)			14 (34.10)		4 (8.30)		
Staging	2.28 ± 1.01	2 (0 to 4)	2.04 ± 0.85	2 (1 to 4)	<0.01	2.27 ± 1.01	2 (0 to 4)	2.17 ± 0.87	2 (1 to 4)	0.60
R Staging										
0	40 (93.00)		83 (97.60)		0.33	40 (93.00)		48 (96.00)		0.66
1	3 (7.00)		2 (2.40)			3 (7.00)		2 (4.00)		
Hospitalization (days)	10.16 ± 7.66	8 (5 to 49)	7.07 ± 3.05	6 (4 to 26)	**<0.01**	10.16 ± 7.66	8 (5 to 49)	7.24 ± 3.51	6 (4 to 26)	**<0.01**
N staging	0.68 ± 0.80	0 (0 to 2)	0.42 ± 0.67	0 (0 to 2)	0.08	0.68 ± 0.80	0 (0 to 2)	0.50 ± 0.71	0 (0 to 2)	0.29
T staging	3.97 ± 0.85	4 (2 to 5)	3.63 ± 0.91	4 (0 to 5)	0.03	3.90 ± 0.86	4 (2 to 5)	3.60 ± 1.03	4 (0 to 5)	0.16
M staging	33 (64.70)		30 (34.50%)		**<0.001**	17 (33.30%)		5 (9.80%)		**<0.001**
Surgery complications (yes)	36 (70.60)		67 (77.00)		0.40	15 (30.00)		10 (19.00)		0.35
Previous surgery (yes)	30 (58.80)		50 (57.70)		0.87	30 (58.80)		27 (52.90)		0.54
Readmission (yes)	6 (11.8)		10 (11.50)		0.96	6 (11.80)		7 (13.70)		0.99
2nd Surgery (yes)	7 (13.70)		10 (21.80)		0.23	7 (13.70)		14 (27.50)		0.08
Comorbidity (yes)	43 (84.30)		71 (81.60)		0.68	43 (84.30)		37 (72.50)		0.14
Harvested LNF (n)	19.09 ± 9.4	17.5 (1 to 41)	16.82 ± 9.75	14.5 (1 to 57)	0.10	19.09 ± 9.4	17.5 (1 to 41)	16.14 ± 10.28	13 (2 to 57)	0.14
Positive LFN(n)	2.18 ± 4.21	0 (0 to 16)	1.13 ± 3.55	0 (0 to 25)	0.24	2.18 ± 4.21	0 (0 to 16)	1.27 ± 4.04	0 (0 to 25)	0.40
Surgery length (time)	140.11 ± 50.8	127.5 (60 to 355)	144.94 ± 42.33	142.5 (60 to 300)	0.33	140.11 ± 50.8	127.5 (60 to 355)	146.4 ± 42.32	147.5 (70 to 270)	0.51
Length of specimen (cm)	34.11 ± 13.8	32 (8.5 to 80)	29.85 ± 10.69	30 (6 to 91)	**0.04**	34.11 ± 13.8	32 (8.5 to 80)	28.21 ± 8.09	27.5 (6 to 55)	**0.01**
Clearance < 1 cm/CRM < 1 mm	4 (8.20)		6 (7.00)		0.80	4 (8.20)		3 (6.00)		0.71
CLAVIEN-DINDO										
*0*	19 (40.40)		51 (60.00)		0.14	19 (40.40)		29 (58.00)		0.36
*I*	5 (10.60)		10 (11.80)			5 (10.60)		6 (12.00)		
*II*	15 (31.90)		19 (22.40)			15 (31.90)		12 (24.00)		
*III*	4 (8.50)		2 (2.40)			4 (8.50)		1 (2.00)		
*IV*	3 (6.40)		3 (3.50)			3 (6.40)		2 (4.00)		
V	1 (2.10)		-			1 (2.10)		-		
Liquid oral diet (days)	2.77 ± 1.2	3 (1 to 6)	1.96 ± 0.99	2 (1 to 7)	**<0.01**	2.77 ± 1.2	3 (1 to 6)	1.98 ± 1.15	2 (1 to 7)	**<0.01**
Solid oral diet (days)	4.41 ± 1.25	4 (3 to 7)	3.45 ± 1.15	3 (2 to 8)	**<0.01**	4.41 ± 1.25	4 (3 to 7)	3.35 ± 1.32	3 (2 to 8)	**<0.01**
Colostomy	6 (11.80)		1 (1.01)		**<0.01**	6 (11.80)		-		**<0.01**
Ileostomy	6 (11.80)		21 (24.10)			6 (11.80)		14 (27.50)		
No Stomy	39 (76.50)		65 (74.70)			39 (76.50)		37 (72.50)		
Time of observation (month)	53.7 ± 29.5	75 (1 to 75)	69.6 ± 15.8	75 (3 to 75)	**<0.01**	53.69 ± 29.5	75 (1 to 75)	70.96 ± 14.82	75 (3 to 75)	
Comorbidity score	2.51 ± 1.86	2 (0 to 7)	1.91 ± 1.42	2 (0 to 6)	0.07	2.51 ± 1.86	2 (0 to 7)	1.75 ± 1.45	2 (0 to 5)	**0.04**
Relapses	3 (6.70)		10 (12.00)		0.54	3 (6.70)		5 (10.40)		0.71
Time to relapse	66.0 ± 0.0	66 (66 to 66)	60.40 ±15.7	66 (5 to 66)	**0.01**	66 ± 0	66 (66 to 66)	59.14 ± 17.54	66 (5 to 66)	**0.01**

All data are shown as mean ± sd, median (min to max) for continuous variables and as n (%) for proportions. *Mann Whitney U test for continuous variables and Chi-squared test/Fisher’s exact test for categorical ones, when appropriate.* Legend: BMI: Body Mass Index; LNF: Lymph Nodes.

**Table 2:** Propensity score matching balance table.

	Type	Diff. Un	M. Threshold	V. Ratio. Adj	V. Threshold
distance	Distance	1.576		0.1172	
Age (years)	Contin.	−1	Not Balanced. >0.1	1.0305	Balanced. <2
Sex (Male)	Binary	0.2941	Not Balanced. >0.1	NA	
BMI (Kg/m^2^)	Contin.	−0.8841	Not Balanced. >0.1	0.1871	Not Balanced >2

Legend: BMI: Body Mass Index.

**Table 3:** Multivariate Cox regression analysis on overall mortality.

	HR	CI 95%	*p*
	raw Model		
Treatment (VL)	0.16	0.05 to 0.48	**<0.01**
	Model 1		
Treatment (VL)	0.28	0.10 to 0.99	**0.05**
Age (years)	1.03	0.98 to 1.09	0.19
Sex (Male)	0.66	0.28 to 1.56	0.34
BMI (kg/m^2^)	1.02	0.94 to 1.11	0.57
	Model 2		
Treatment (VL)	0.28	0.10 to 0.99	**0.05**
Age (years)	1.03	0.98 to 1.10	0.19
Sex (Male)	0.66	0.98 to 1.10	0.35
BMI (kg/m^2^)	1.02	0.94 to 1.11	0.59
Location (rectum)	0.99	0.38 to 2.57	0.99
Location (left)	1.01	0.33 to 3.11	0.97
	Model 3		
Treatment (VL)	0.03	0.02 to 0.37	**<0.01**
Age (years)	0.97	0.88 to 1.07	0.63
Sex (Male)	0.48	0.06 to 4.42	0.52
BMI (kg/m^2^)	0.97	0.76 to 1.24	0.85
Location (rectum)	0.31	0.04 to 2.29	0.25
Location (left)	7.53	0.67 to 83.93	0.10
Length of specimen	0.97	0.92 to 1.02	0.37
Previous surgery	1.54	0.22 to 10.48	0.65
Staging	2.81	0.94 to 8.43	0.06
Clearance < 1 cm/CRM < 1 mm	3.02	0.19 to 45.81	0.42
Nodes (n)	1.04	0.88 to 1.23	0.61
Comorbidity score	1.09	0.61 to 1.93	0.76

Legend: BMI: Body Mass Index; VL: Video laparoscopic surgery.

**Table 4:** Cox regression analysis on tumor relapse.

	HR	CI 95%	*p*
	raw Model		
Treatment (VL)	1.70	0.40 to 7.13	0.46
	Model 1		
Treatment (VL)	1.38	0.50 to 38.84	0.23
Age (years)	1.03	0.97 to 1.13	0.52
Sex (Male)	0.37	0.07 to 1.74	0.21
BMI (kg/m^2^)	1.03	0.86 to 1.24	0.70
	Model 2		
Treatment (VL)	1.42	0.42 to 40.00	0.22
Age (years)	1.03	0.93 to 1.13	0.51
Sex (Male)	0.40	0.08 to 2.00	0.26
BMI (kg/m^2^)	1.04	0.87 to 1.24	0.64
Location (rectum)	1.32	0.21 to 8.09	0.75
Location (left)	0.79	0.21 to 8.01	0.82
	Model 3		
Treatment (VL)	3.69	0.32 to 41.29	0.28
Age (years)	1.02	0.93 to 1.13	0.58
Sex (Male)	2.31	0.08 to 2.18	0.30
BMI (kg/m^2^)	1.03	0.85 to 2.17	0.66
Location (rectum)	1.26	0.20 to 7.81	0.80
Location (left)	0.76	0.09 to 6.15	0.80
Staging	1.19	0.55 to 2.56	0.64

Legend: BMI: Body Mass Index; VL: Video laparoscopic surgery.

## Data Availability

The data presented in this study are available on request to the corresponding author.
